# *CCR2-V64I *polymorphism is associated with increased risk of cervical cancer but not with HPV infection or pre-cancerous lesions in African women

**DOI:** 10.1186/1471-2407-10-278

**Published:** 2010-06-10

**Authors:** Koushik Chatterjee, Collet Dandara, Margaret Hoffman, Anna-Lise Williamson

**Affiliations:** 1 Division of Medical Virology and Institute of Infectious Disease and Molecular Medicine (IIDMM), University of Cape Town, Cape Town, Republic of South Africa; 2 Division of Human Genetics, Faculty of Health Sciences, University of Cape Town, Cape Town, Republic of South Africa; 3 School of Public Health and Family Medicine, University of Cape Town, Cape Town, Republic of South Africa; 4 National Health Laboratory Service, Groote Schuur Hospital, Observatory, Cape Town, Republic of South Africa

## Abstract

**Background:**

Cervical cancer, caused by specific oncogenic types of human papillomavirus (HPV), is the second most common cancer in women worldwide. A large number of young sexually active women get infected by HPV but only a small fraction of them have persistent infection and develop cervical cancer pointing to co- factors including host genetics that might play a role in outcome of the HPV infection. This study investigated the role of *CCR2-V64I *polymorphism in cervical cancer, pre-cancers and HPV infection in South African women resident in Western Cape. *CCR2-V64I *polymorphism has been previously reported to influence the progression to cervical cancer in some populations and has also been associated with decreased progression from HIV infection to AIDS.

**Methods:**

Genotyping for *CCR2-V64I *was done by PCR-SSP in a case-control study of 446 women (106 black African and 340 mixed-ancestry) with histologically confirmed invasive cervical cancer and 1432 controls (322 black African and 1110 mixed-ancestry) group-matched (1:3) by age, ethnicity and domicile status. In the control women HPV was detected using the Digene Hybrid Capture II test and cervical disease was detected by cervical cytology.

**Results:**

The *CCR2-64I *variant was significantly associated with cervical cancer when cases were compared to the control group (P = 0.001). Further analysis comparing selected groups within the controls showed that individuals with abnormal cytology and high grade squamous intraepitleial neoplasia (HSIL) did not have this association when compared to women with normal cytology. HPV infection also showed no association with *CCR2-64I *variant. Comparing SIL positive controls with the cases showed a significant association of *CCR2-64I *variant (P = 0.001) with cervical cancer.

**Conclusions:**

This is the first study of the role of *CCR2-V64I *polymorphism in cervical cancer in an African population. Our results show that *CCR2-64I *variant is associated with the risk of cervical cancer but does not affect the susceptibility to HPV infection or HSIL in South African women of black and mixed-ancestry origin. This result implies that the role of CCR2 is important in invasive cancer of the cervix but not in HPV infection or in the development of pre-cancers.

## Background

Cervical cancer is the second leading cause of cancer and cancer related mortality in women worldwide with 493,243 cases and 273,505 deaths per year [1]. The incidence and mortality rates have dropped significantly in developed countries due to effective cervical screening programs thus 83% of all new cervical cancer cases and 85% of all cervical cancer-related deaths now occur in developing countries where screening programs are poor [2]. Cervical cancer is the most common cancer and the highest cause of cancer death in women in sub-Saharan Africa accounting for 22% of all cancers in women [3].

Squamous cell carcinoma of the cervix arises from the pre-cancer lesions of squamous intraepithelial lesions (SIL) [4] which are further divided into low-grade (LSIL) and high-grade (HSIL) stages. LSILs are pre-cancerous and considered as the very early precursor stages of cervical cancer with very few cases progressing to cancer [5] while most HSILs progress to invasive cervical cancer (ICC) when left untreated [6]. Cervical cancer is a multistep process that develops slowly over several years after persistent infection of epithelial cells with oncogenic types of human papillomavirus (HPV), mainly types 16 and 18. Many women become infected with HPV during their life-time but most are able to clear the infection. Less than 1% of the clinically detectable HPV infections progress to cervical cancer [7]. This indicates that additional risk factors play an important role in the development of the cancer of the cervix. These risk factors include host and viral genetic factors as well as environmental and life style factors [8]. The possibility of genetic predisposition is strengthened by the observation of a double risk of developing cervical cancer in biological first degree relatives [9] with these factors most likely influencing response to HPV infection and its persistence. The involvement of macrophages, natural killer (NK) cells and T-cells in papilloma regression further point to the role of genes associated cell-mediated immunity (CMI) in the risk of infection and persistence of HPV [8]. Data showing increased incidence of SIL in immusuppressed women and in immunocompromised patients as well as in immunomanipulated animal models show a strong role of the immune system in controlling the infection [7,10].

One of the earliest responses of human body to injury or infection is the release of chemokines that triggers recruitment of local inflammatory and immune cells. Chemokines are chemoattractant cytokines that regulate migration of leukocytes by binding to G-protein coupled cell-surface receptors. It has been postulated that HPV disrupts the interaction between epithelial cells and the immune system by deregulating the expression of chemokines which can be mediated by (HPV) E7 which interacts with IRF-1 [11].

Chemokine receptor 2 or CCR2, has affinity for CCL2 (monocyte chemoattractant protein-1 (MCP-1)), CCL7, CCL8 and CCL13 ligands and is expressed on basophils, monocytes, dendritic cells (DCs), activated T-cells and NK cells. CCR2 is a major receptor for the MCP-1 which is produced largely by tumour cells [12,13] and is responsible for recruiting macrophages to tumours in bladder, cervix, ovary, lung and breast. The role of macrophages in tumour development and control is multifactorial. In early stages, macrophages have tumour cytotoxic characteristics but once tumour cells have evaded the immune system they switch to play a role in tumour angiogenesis [14-16]. A pilot study done by Riethdorf et al. (1996) showed that 1/6 high grade squamous intraepithelial neoplasia expressed MCP-1 compared to 4/5 squamous cell cervical caracinomas [17] suggesting that MCP-1 plays a greater role in invasive cancers compared to pre-cancers. This was further confirmed by *in situ *hybridisation studies [18] where MCP-1 expression was detected in the stoma surrounding the tumour particularly at the invasion front.

CCR2 gene is localized on chromosome 3p21 within a cluster of chemokine receptor genes. It has two isoforms; CCR2A and CCR2B products of the CCR2 gene as a result of alternative splicing. A single nucleotide polymorphism (SNP) of G to A at position 190 of CCR2 gene changes amino acid valine (GTC) to isoleucine (ATC) at codon 64 (*CCR2-V64I*). This conservative amino acid change takes place in the first transmembrane domain of CCR2A and CCR2B [19]. This change makes CCR2A more stable and increases its half-life but does not in any way affect the stability of CCR2B isoform [19]. The *CCR2-V64I *polymorphism has been extensively studied and several reports show an association of the *CCR2-64I *variant with reduced risk of progression to AIDS in HIV-infected individuals, [20-23], multiple sclerosis [24] and development of breast cancer [25]. It is also associated with increased risk of carotid atherosclerosis [26] and reduction in the risk of renal transplant rejection [27].

There are conflicting reports on the role of the *CCR2-V64I *polymorphism in the development or risk to cervical cancer [28-31]. A study comparing SIL patients to ICC patients in a Portuguese population reported that the *CCR2-64I *variant was associated with reduced risk of developing ICC from HSIL [29]. The same group conducted a case-control study in the same population comparing women with HSIL to a control group and found *CCR2-64I *variant as a risk allele for developing HSIL [28]. Among two studies in Swedish population the first study [30] reported that the *CCR2-64I *variant was associated with decreased risk of developing cervical cancer. The second Swedish study did not find any association of *CCR2-64I *variant with either HPV infection or cancer of the cervix [31].

Epidemiological studies have demonstrated the importance of host genetic susceptibility factors in the development of cervical cancer. A twin study [32] and a mother-daughter family study [33] demonstrated a hereditary component of cervical tumours. Biological first-degree relatives of women with cancer of the cervix showed an increased risk of developing cervical cancer compared to non-biological relatives of women with cervical cancer [9]. There are conflicting reports on the role of the *CCR2-V64I *polymorphism in cervical cancer and furthermore, none of these studies have been done in African population, thus, we therefore investigated the role of *CCR2-V64I *polymorphism in risk of cancer of the cervix in South African women of black and mixed-ancestry origin.

## Methods

### Participants

A total of 1878 subjects comprising 446 women with invasive cervical cancer (106 black African and 340 women of mixed-ancestry) and 1432 controls (322 black African and 1110 women of mixed-ancestry) without cancer of the cervix were recruited in a cross-sectional study. Incident cases of symptomatic invasive cervical epithelial cancer (stage 1b-IVb), diagnosed a maximum of six months previously were recruited from Groote Schuur Hospital and Tygerberg Hospital in the Western Cape Province, South Africa. Hospital controls were series matched in a ratio of 3:1 to the cases on decade of age, ethnic group and area of residence (urban/rural). Most of the Black African subjects originally came from either the Transkei, in the Eastern Cape of South Africa while their Mixed Ancestry group refers to a group of people that show highest level of intercontinental admixture of any global population resulting from gene flow between Black Africans, Western Europeans, Khoisans, Indians, East Asians (eg. Malaysians) and Cushitics who settled in the Western Cape region [34]. There were 264 (59%) urban cases and 182 (41%) rural cases compared to 718 (53%) urban controls and 632 (47%) rural controls (residency status was not known for 82 controls). The cases and controls formed part of a study to investigate the association of oral contraceptives with cervical cancer [35,36].

The mean age for black cases was 43.8 yrs (SD 9.2) and for mixed-ancestry cases it was 46.0 yrs (SD 8.1). The mean age for black controls was 42.3 yrs (SD 9.0) and for mixed-ancestry controls it was 44.3 yrs (SD 8.4). The HIV infection status was 4.8% for the cases and 4.7% for the controls. No significant differences in age or HIV status were observed between cases and controls (data not shown here). A significant difference in the distribution of the smoking status was found between the cases and the controls (P = 0.004) (data not shown here). Subsequently, all the analyses were adjusted for the smoking status along with ethnicity. The observed genotype frequencies for *CCR2-V64I *polymorphism in the controls were not in Hardy-Weinberg equilibrium (P = 0.001) (data not shown here). This could be due to the fact the our controls were not a randomly chosen population rather handpicked in a 3:1 matched ratio to the cases on decade of age, ethnic group and area of residence (urban/rural).

### Clinical specimens

Blood was collected from cases and controls, following written informed consent and stored at -80°C. The genetic study was approved by the University of Cape Town Human Ethics Committee (REC REF: 075/2009). The subject identifiers were permanently unlinked from the specimens.

### Papanicolaou test

Endocervical scrapings were taken from the control women to conduct papanicolaou test (Pap smear) to check for their cytology status as previously described [35]. For this paper samples positive for ASCUS (atypical squamous cells of undetermined significance), LSIL or HSIL were considered as abnormal cytology.

Among 1258 controls for which pap smear results were available, 185 (15%) were positive. Of the 185 pap smear positive samples, 91 (49%) were classified as SIL and the remaining 94 (51%) were classified as ascus (Atypical Squamous Cells of Undetermined Significance) positive. Among those 91 SIL positive samples, 46 (51%) were positive for LSIL and 45 (49%) were positive for HSIL.

#### High-risk HPV type detection

Endocervical scrapings from control women were assayed for HPV infection using the Digene Hybrid Capture II HPV Test for the detection of high risk HPV types 16/18/31/33/35/39/45/51/52/56/58/59/68 and were classified positive according to the manufacturer's instructions (Digene Corporation, Gaithersburg, MD, USA) [35].

### Extraction of genomic DNA

The genomic DNA was extracted using TotalNucleicAcid Extraction kit for MagNA Pure Compact nucleic acid extractor (Roche Diagnostics, Germany).

### Determination of CCR2-V64I polymorphism

The *CCR2-V64I *polymorphism was determined by polymerase chain reaction (PCR) using sequence-specific primers (SSP) followed by agarose gel electrophoresis. Two PCR reactions were carried out for each sample using two different forward primers (CCR2-64V and CCR2-64I) and a reverse primer. 130 ng of genomic DNA was amplified in a 10-μl reaction mixture containing 10 picomoles of each CCR2-V64I primers: F(CCR2-64V), 5'TGGGCAACATGCTGGTCG3' or F(CCR2-64I), 5'TGGGCAACATGCTGGTCA3' and R, 5'TGGAAAATAAGGGCCACAGAC3' [37] and 5-μl 2 × ImmoMix™ (Bioline). PCR cycle reactions were performed on an ABI 2720 Thermal Cycler (Applied Biosystems, Foster City, CA) beginning with a denaturing step at 95°C for 2.5 min followed by 10 higher-stringency cycles of denaturing at 94°C for 25 s, annealing at 60°C for 45 s and extension at 72°C for 45 s again followed by 21 lower-stringency cycles of denaturing at 94°C for 25 s, annealing at 58°C for 40 s and extension at 72°C for 40 s with a final extension at 72°C for 6 min. The PCR reaction conditions were adapted and modified from Tang J et. al. [37].

**Figure 1:**
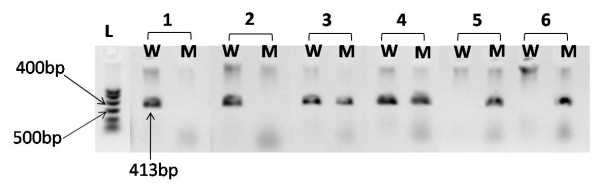
**Analysis of the *CCR2-V64I *genotypes on agarose gel**. L = DNA ladder, samples 1 and 2 = GG (CCR2-64V - wild type homozygous), samples 3 and 4 = GA (CCR2-V64I - heterozygous), samples 5 and 6 = AA (CCR2-64I - mutant type homozygous), W = PCR amplifying wild type variant (CCR2-64V), M = PCR amplifying mutant type variant (CCR2-64I).

The amplified PCR products of 413 base pairs (bp) were analysed by running on 1.5% agarose gel stained with ethidium bromide using an O'GeneRuler™ 50 bp DNA Ladder, ready-to-use (Fermentas Inc, Ontario, Canada). The samples with a wild type allele (G) at position 190 of the CCR2 gene amplified the PCR containing CCR2-64V forward primer and showed no products for the PCR containing CCR2-64I forward primer. Likewise, samples with a mutant allele (A) at position 190 of the CCR2 gene did not show any product for the PCR containing CCR2-64V forward primer rather amplified the PCR containing CCR2-64I forward primer. The heterozygous samples showed products for both the PCR containing CCR2-64V as well as CCR2-64I forward primers (Figure 1).

The genotyping was cross-checked by DNA sequencing of total 40 samples, 10 each for wild type, mutant type, heterozygous and 10 randomly selected samples using a forward primer (5'TACGGTGCTCCCTGTCATAAA3') and the same reverse primer previously used for genotyping of CCR2. The DNA sequencing was done using a BigDye Teminator V3.1 Cycle sequencing kit (Applied Biosystems, Foster City, CA) following the manufacturers protocol. Other than the mutant type samples, all the other samples showed 100% confirmation of our genotype results. Due to some ambiguity in the first 10 randomly selected mutant type samples, we decided to sequence all the mutant samples. Among all the 125 mutant samples (as assigned according to PCR amplification and agarose gel results) only 3 did not show any result in both forward and reverse primer sequencing and were excluded from the final analysis. Rest of the 122 samples showed clear sequencing results of GG, AG or AA and were scored accordingly.

### Statistical Analysis

The genotype distributions were tested for Hardy-Weinberg equilibrium in cases and controls. Logistic regression was used to test for genotype associations with cervix cancer status as well as baseline characteristics (age, ethnicity and smoking status) and secondary outcomes such as HIV status and abnormal cytology (Pap smear) in the control group. Statistical analyses were done using Stata 9 software.

## Results

The role of *CCR2-V64I *polymorphism was investigated in the risk of cancer of the cervix, pre-cancers and HPV infection in South African women of black and mixed-ancestry origin.

Genotyping data for *CCR2-V64I *polymorphism was obtained on 1378 of the 1432 control specimens. The frequency of *CCR2-64I *variant was 39% in black cases and 20% in the black controls and 40% in mixed-ancestry cases and 18% in mixed-ancestry controls. A statistically significant, association was found with *CCR2-64I *variant (P = 0.001) and cervical cancer cases and controls after adjusting for ethnicity and smoking (Table 1).

**Table 1:** Counts (n), frequencies (%) and association statistics for *CCR2-V64I *genotypes for cases and controls

	Controls (n = 1378)		Cases (n = 446)			
	Black 305 (n = 22)	Mixed-ancestry 1073 (n = 78)	Black 106 (n = 24)	Mixed-ancestry 340 (n = 76)	Genotype-cervical cancer association, adjusted for ethnicity and smoking	
**Genotypes**	**n (%)**	**n (%)**	**n (%)**	**n (%)**	**P-value**	**OR (95% CI)**
**CCR2 G**→**A**						
GG	189 (62)	704 (66)	24 (23)	78 (23)	-	1
AG	112 (37)	356 (33)	81 (76)	255 (75)	0.001	6.22 (4.85-7.97)
AA	4 (1)	13 (1)	1 (1)	7 (2)	0.002	3.99 (1.68-9.50)
AG + AA	116 (38)	369 (34)	82 (77)	262 (77)	0.001	6.14 (4.79-7.86)

P-values and OR (95% confidence intervals) are for test of genotype or additive allelic association with cervix cancer risk, adjusted for ethnicity and smoking. P-values next to genotype names are for joint model, others are for ORs of specific genotype compared to reference genotype, indicated with OR = 1. Cases = Women with cancer of the cervix (ICC), Controls = Women without cancer of the cervix.

Since cervical cancer is a multi-step process which starts with HPV infection it was of interest to determine at which stage the *CCR2-64I *variant became significant. Therefore the *CCR2-64I *variant was also studied in women with cervical pre-cancers and HPV infection. When the SIL positive (LSIL and HSIL positive) controls (n = 91) were compared with the invasive cervical cancer cases (n = 446) for *CCR2-V64I *polymorphism, a statistically significant association was found with the presence of *CCR2-64I *variant (P = 0.001) in the invasive cancers adjusted for ethnicity and smoking (Table 2). This result indicated a difference in the *CCR2-64I *variant of women who developed invasive cancer compared to the women with pre-cancers.

**Table 2:** Association statistics for *CCR2-V64I *genotypes for cases and SIL positive controls

	SIL positive controls (n = 91)		Cases (n = 446)			
	Black 31 (34)	Mixed-ancestry 60 (66)	Black 106 (24)	Mixed-ancestry 340 (76)	Genotype-cervical cancer association, adjusted for ethnicity and smoking	
**Genotypes**	**n (%)**	**n (%)**	**n (%)**	**n (%)**	**P-value**	**OR (95% CI)**
**CCR2 G**→**A**						
GG	21 (68)	40 (66)	24 (23)	78 (23)	-	1
AG	9 (29)	19 (32)	81 (76)	255 (75)	0.001	7.18 (4.35-11.87)
AA	1 (3)	1 (2)	1 (1)	7 (2)	0.300	2.32 (0.47-11.36)
AG + AA	10 (32)	20 (33)	82 (77)	262 (77)	0.001	6.86 (4.19-11.21)

Cases = Women with cancer of the cervix (ICC), SIL positive controls = Women without cancer of the cervix (ICC) but positive for LSIL and HSIL by pap smear test.

Since the association was found with cancer it was then determined if the same association could be observed in pre-cancers within the control group. The association of *CCR2-V64I *polymorphism with abnormal cytology status, HSIL status and high risk HPV status was investigated in the control group. Both abnormal cytology (P = 0.437) (Table 3) and high risk HPV status (P = 0.913) (Table 4) were not found to be associated with *CCR2-64I *variant in the control group adjusted for ethnicity and smoking. Comparing only the HSIL positive samples with all the normal cytology samples also did not show any significant association (P = 0.157) in controls adjusted for ethnicity and smoking (Table 5).

**Table 3:** Association statistics for *CCR2-V64I *genotypes according to cytology in the control group

	Normal cytology (n = 1070)		Abnormal cytology (n = 185)			
	Black 210 (20)	Mixed-ancestry 860 (80)	Black 64 (35)	Mixed-ancestry 121 (65)	Genotype-abnormal cytology association, adjusted for ethnicity and smoking	
**Genotypes**	**n (%)**	**n (%)**	**n (%)**	**n (%)**	**P-value**	**OR (95% CI)**
**CCR2 G**→**A**						
GG	124 (59)	563 (66)	45 (70)	78 (64)	-	1
AG	84 (40)	287 (33)	17 (27)	40 (33)	0.290	0.83 (0.59-1.17)
AA	2 (1)	10 (1)	2 (3)	3 (3)	0.134	2.28 (0.78-6.69)
AG + AA	86 (41)	297 (35)	19 (30)	43 (36)	0.437	0.88 (0.63-1.22)

Abnormal cytology = Positive for pap smear test (i.e. positive for ASCUS + positive for LSIL + positive for HSIL), Normal cytology = Negative for pap smear test.

**Table 4:** Association statistics for *CCR2-V64I *genotype for high risk HPV infection in the control group

	High risk HPV positive (201)		High risk HPV negative (1053)			
	Black 62 (31)	Mixed-ancestry 139 (69)	Black 212 (20)	Mixed-ancestry 841 (80)	Genotype-high risk HPV association, adjusted for ethnicity and smoking	
**Genotypes**	**n (%)**	**n (%)**	**n (%)**	**n (%)**	**P-value**	**OR (95% CI)**
**CCR2 G**→**A**						
GG	34 (55)	94 (68)	135 (64)	546 (65)	-	1
AG	27 (43)	41 (29)	74 (35)	286 (34)	0.904	0.98 (0.71-1.35)
AA	1 (2)	4 (3)	3 (1)	9 (1)	0.163	2.14 (0.73-6.25)
AG + AA	28 (45)	45 (32)	77 (36)	295 (35)	0.913	1.02 (0.74-1.40)

High risk HPV positive = Positive for Hybrid Capture II HPV Test, High risk HPV negative = Negative for Hybrid Capture II HPV Test.

**Table 5:** Association statistics for *CCR2-V64I *genotypes for HSIL in the control group

	**Normal cytology **(n = 1070)		**HSIL positive **(n = 45)			
	Black 210 (20)	Mixed-ancestry 860 (80)	Black 16 (36)	Mixed-ancestry 29 (64)	Genotype-HSIL association, adjusted for ethnicity and smoking	
**Genotypes**	**n (%)**	**n (%)**	**n (%)**	**n (%)**	**P-value**	**OR (95% CI)**
**CCR2 G**→**A**						
GG	124 (59)	563 (66)	12 (75)	21 (72)	-	1
AG	84 (40)	287 (33)	4 (25)	8 (28)	0.190	0.64 (0.32-1.25)
AA	2 (1)	10 (1)	0 (0)	0 (0)	-	-
AG + AA	86 (41)	297 (35)	4 (30)	8 (28)	0.157	0.61 (0.31-1.21)

HSIL positive = Positive for HSIL by pap smear test, Normal cytology = Negative for pap smear test.

## Discussion

Polymorphisms in the CCR2 gene that alters the macrophage recruitment have been reported to influence a number of diseases including AIDS [20-23], multiple sclerosis [24], breast cancer [25], carotid atherosclerosis [26] and renal transplant rejection [27]. This polymorphism has also been associated with cervical cancer in two different populations [28-31].

Persistent HPV infection of epithelial cells is necessary for the carcinogenesis of the uterine cervix, but not all HPV infected cervical lesions progress to cervical cancer. In addition many pre-cancers do not progress to invasive cancer and can regress. Chemokines are regarded as an important cofactor in the progression of the cervical lesions to cancer of the cervix [7].

We are the first to study the frequency of the *CCR2-V64I *polymorphism in an indigenous black African as well as in a mixed-ancestry population. We found a statistically significant association of *CCR2-64I *variant (P = 0.001) with cervical cancer in African black and mixed-ancestry women adjusted for ethnicity and smoking. Our data suggests that women carrying A allele (P = 0.001, OR (95% CI) = 6.14 (4.79-7.86)) and A/A genotype (P = 0.002, OR (95% CI) = 3.99 (1.68-9.50)) at position 190 of the CCR2 gene have increased risk of cervical cancer compared to women carrying the G variant of it. When the SIL controls were compared with cervical cancer cases, it was found that *CCR2-64I *carriers are at more risk of developing cervical cancer (P = 0.001, OR (95% CI) = 6.86 (4.19-11.21)) from SIL. The analysis of *CCR2-V64I *genotypes with abnormal cytology (P = 0.437), presence of high risk HPV infection (P = 0.913) and HSIL positive status (P = 0.157) did not show any statistically significant association in the control group.

**Figure 2:**
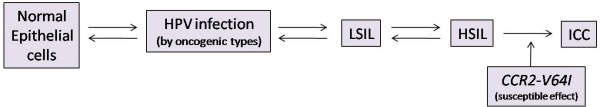
**A schematic diagram showing the susceptible effect of CCR2-64I variant during development of ICC**.

Our results showing susceptible effect of *CCR2-64I *variant to cervical cancer (compared to controls without cancer of the cervix and SIL positive controls) do not agree with those of two other groups, Coelho et al. [29] and Ivansson et al [30]. Also our data showing no association of *CCR2-64I *variant with HSIL when compared to individuals with normal cytology, do not confirm Coelho et. al. [28] who reported an increased risk of *CCR2-64I *variant with HSIL. The contradictory results might be due to one or several factors, including the difference in ethnic origin of the populations studied the difference in sample size and the high percentage of the mutant allele (*CCR2-64I*) in our population. The fact that we did not find any association with abnormal cytology, HSIL and high risk HPV infection in the control group but found a susceptible effect with cervical cancer suggests that *CCR2-64I *variant is not associated with susceptibility to HPV infection and pre-cancerous lesions in our population but increases the risk of ICC at a later stage during the development of cancer of the cervix from HSIL (Figure 2). Our results showing no association of *CCR2-64I *variant with HPV infection are in line with the findings of Zheng B et. al [31].

HPV-infected epithelial cells do not elicit strong local or systemic immune responses. MCP-1 plays an important role in the development of tumours as it is one of the major chemokines that induces recruitment of macrophages in tumours including cervical cancer [14]. Recruitment and activation of macrophages is a vital process for the inflammatory response of the human body. Though macrophages display tumour cytotoxicity, tumour-associated macrophages (TAMs) mainly have protumour functions [38] and help in tumour angiogenesis. Increased expression of MCP-1 recruits more macrophages which speed up the process of tumour destruction or progression depending upon the type of macrophages recruited. After the infection of epithelial cells by HPV, the MCP-1 expression decreases from LSIL to HSIL and increases again from HSIL to ICC [17]. Tumour cells have been reported with high levels of MCP-1 expression [18]. Macrophages which are recruited by MCP-1 chemokine, express CCR2 on their cell surface. The *CCR2-64I *variant is associated with increased expression of CCR2A (due to increased stability of CCR2A) on the cell surface of monocytes. This increases the attraction of monocytes to tumour cells producing MCP-1. In early stages of infection, the increased recruitment of monocytes results in more macrophages and associated cells (DC and NK cells) converging on the developing tumours to destroy the progressing tumours cells. Therefore, increased expression of CCR2 receptors results in increased recruitment of macrophages and possibly faster destruction of a developing tumour. Thus the *CCR2-64I *variant might be associated with reduced risk of developing cancer in the early phase. Our results with HPV infected individuals do point toward a reduced risk of *CCR2-64I *with abnormal cytology and HSIL positivity when compared to individuals with normal cytology (according to the tendencies of the ORs) though not statistically significant.

However, once the tumour cells evade the immune system, the macrophages that are recruited towards elimination of the tumour switch to TAMs [15,16]. The increased stability of CCR2A due to *CCR2-64I *variant sustains the tumours by continuously recruiting TAMs that support tumour angiogenesis. It is not known when this switch from tumour cytotoxic macrophages to TAMs occurs during the development of cervical cancer. We hypothesize that the switch occurs during early phase of HSIL. Thus the *CCR2-64I *variant would be associated with increased risk of cancer in the later stage of tumour development (in this situation after progressing to HSIL) when compared to the *CCR2-64V *variant which is associated with less stable CCR2A stability and expression.

## Conclusions

Our study showed a significant association of *CCR2-V64I *polymorphism with cervical cancer, but did not show any association with HPV infection and pre-cancerous lesions. *CCR2-64I *variant showed an increased risk of cervical cancer but not with infection by HPV and pre-cancerous lesions. This implies that this mutation is associated with a late event in the progression to cervical cancer. Further studies are needed with patients in different stages of cervical cancer to confirm our findings.

## Competing interests

The authors declare that they have no competing interests.

## Authors' contributions

KC participated in planning of the study, performed extraction of DNA, genotyping, analyzing the results and advanced statistical analysis. CD helped in statistical analysis and interpreting of the results. MH coordinated the collection of the biological samples. ALW was responsible for the storage of the biological samples, helped in planning and supervising the study and helped in interpretation of the results. All authors critically read and took part in finalizing the manuscript.

## Pre-publication history

The pre-publication history for this paper can be accessed here:

http://www.biomedcentral.com/1471-2407/10/278/prepub
